# “And in some cases, we're the best option:” A qualitative study of community‐based doula support for black perinatal mental health

**DOI:** 10.1002/ajcp.70020

**Published:** 2025-09-30

**Authors:** Tamara Nelson, Naysha N. Shahid, Samrawit B. Gebretensay, Chareina C. Johnson, Penny D. Telesford, Karen Sheffield‐Abdullah

**Affiliations:** ^1^ Department of Psychology Rutgers University Camden Camden USA; ^2^ Department of Psychology University of Miami Camden USA; ^3^ Department of Prevention Science Rutgers University Camden Camden USA; ^4^ School of Nursing University of North Carolina at Chapel Hill Chapel Hill USA

**Keywords:** Black perinatal mental health, community‐based doulas, perinatal mental health screening

## Abstract

We explored community‐based doulas' perspectives on the acceptability of using formal screening tools to address low rates of mental health screening, diagnosis, and treatment for perinatal anxiety and depression among Black women. Using thematic analysis, we analyzed interview data from 30 community‐based doulas who support Black families during pregnancy, birth, and the postpartum period. Approximately 57% of community‐based doulas supported mental health screening; 23% opposed, and 20% were unsure of whether or not to screen. Four themes emerged from participants' responses, including (1) rethinking screening approaches and procedures; (2) cultural and contextual screening; (3) community‐based doula roles; and (4) client mistrust of mental health questionnaires. Additionally, there were four themes that highlighted community‐based doulas' perspectives of how they might advocate for this population, including (1) bridge to mental health; (2) identification and normalization of symptoms; (3) systemic issues; and (4) mental health specialization. Partnering with trusted community‐based doulas might improve perinatal mental health screening and prevention efforts for Black women experiencing perinatal anxiety and depression.

## Highlights

- Black women are less likely to be screened, diagnosed, and treated for perinatal mental health disorders.
- Community‐based doulas may play a critical role in perinatal mental health care.
- More than half of community‐based doulas endorsed using validated screening tools with their Black perinatal clients.
- Acceptability for screening was underscored by rethinking screening approaches, attending to culture and context, considering community‐based doula roles, and understanding client mistrust.
- Community‐based doula‐identified support included identification and normalization of symptoms, bridging to mental health treatment, and obtaining a mental health specialization.

Due to longstanding structural inequities, Black women experience more stress, trauma, socioeconomic inequality, limited access to healthcare, and less social support, which may increase their risk for perinatal anxiety and depression (Cavanaugh & Nelson, 2022; Crear‐Perry et al., 2021; Pao et al., 2019; Somerville et al., 2021). Despite national recommendations for increased screening for perinatal anxiety and depression, Black women are less likely to be screened, diagnosed, and treated for these conditions (Declercq et al., 2022; Geier et al., 2015; Lara‐Cinisomo et al., 2018; Sidebottom et al., 2021). Recent studies indicate that Black women are at high risk of perinatal anxiety and depression (Declercq et al., 2022; Hernandez et al., 2022; Sujan et al., 2023; Wenzel et al., 2021), which are known risk factors for pregnancy‐related morbidity and mortality (Grote et al., 2010; Shahhosseini et al., 2015). Gendered racism, negative birth experiences, and adherence to idealized mothering, and gendered racialized roles (i.e., Superwoman Schema) in response to such experiences, may also heighten the risk for perinatal anxiety and depression (Clarke et al., 2022; Giscombé & Lobel, 2005; Keefe et al., 2017, 2018; Mehra et al., 2020; Nelson, Tomi, et al., 2023; Nelson, Ernst, et al., 2023; Nelson, Brown, et al., 2023; Rehbein et al., 2024; Rosenthal & Lobel, 2020; Woods‐Giscombé, 2010). Moreover, there is an urgent need for more effective approaches to identify and connect Black women with mental health treatment.

Alarmingly, perinatal mental health treatment rates are low. In a national study, 54% of Black women reported symptoms of prenatal depression; however, when compared to White women, only one‐quarter of Black women were likely to have received postpartum treatment (Declercq et al., 2022). In part, cultural mistrust of the mental health care system and providers, discrimination, stigma, inaccessible mental health care, and spiritual practices explain low mental health treatment utilization (Keefe et al., 2016a, 2016b; Mehra et al., 2020; Nelson et al., 2020; Poleshuck et al., 2013). Black women have also described perinatal mental health treatment as insufficient, ineffectual, and culturally insensitive (Keefe et al., 2016b; Leis et al., 2011). Consequently, Black perinatal mental health care stakeholders have advocated for “reimagining the mental health care landscape” (Matthews et al., 2021). Avenues to achieve equitable and culturally responsive mental health treatment included training and educating providers; supporting Black mental health providers and community‐based organizations led by Black women; respecting and supporting community and traditional healing practices; and championing integrated care models and shared decision‐making (Matthews et al., 2021). In this study, we focused on the role of community‐based doulas.

## Understanding traditional doula and community‐based doula support

A traditional doula is a trained professional who provides ongoing physical, emotional, and informational support to their client before, during, and shortly after birth (DONA International, 2025). Traditional doulas primarily work independently within a private practice framework, serving clients who pay for their services (Bey et al., 2019). Having a doula has been associated with fewer perinatal complications, increased breastfeeding rates, and reduced likelihood of postpartum depression (Bohren et al., 2017; Kozhimannil et al., 2013, 2016). Further, there is emerging evidence that doulas are addressing mental health and substance use challenges experienced by their clients (Haiman et al., 2024, 2025; Quiray et al., 2024).

For instance, Haiman et al. (2025) utilized Kozhimannil et al.'s (2016) maternal health framework of connectedness, agency, respect, knowledge, and potential security to investigate how doulas identify and respond to perinatal mental health and substance use challenges in a qualitative study. Most doulas in this study reported actively inquiring about mental health and substance use and viewed such engagement as an integral component of their role (Haiman et al., 2025). Similarly, in a separate qualitative study, Quiray et al. (2024) found that doulas described a range of practices, including referrals to mental health services, the use of informal screening questions, and the application of standardized tools to assess perinatal anxiety and depression. However, specific information and contextual factors shaping their use were not provided. Notably, in both qualitative studies, doulas represented diverse racial and ethnic backgrounds and reported serving multicultural, multilingual, rural, and socioeconomically diverse populations (Haiman et al., 2025; Quiray et al., 2024).

Community‐based doulas are trusted, skilled, nonmedical professionals who provide continuous physical, emotional, and social support before, during, and after childbirth, including labor, and the postpartum period (Bey et al., 2019; Earls, 2014). Deeply committed to serving marginalized communities, community‐based doulas frequently share cultural backgrounds and lived experiences with their clients, which facilitates trust (Bey et al., 2019; Earls, 2014; Kathawa et al., 2022) Community‐based doulas prioritize birth equity and reproductive justice through education, advocacy, and comprehensive doula services to clients at low or no cost (Bakst et al., 2020; Bey et al., 2019). Their scope of practice generally includes increased prenatal and postpartum home visits; collaboration with healthcare and social service providers, such as transportation, housing services, and the Special Supplemental Nutrition Program for Women, Infants, and Children (WIC) assistance; and continuous supervision, mentorship, and professional development (Bey et al., 2019). In addition, community‐based doulas are trained in culturally responsive, trauma‐informed care grounded in social justice frameworks, enabling them to support diverse clients facing systemic barriers and limited social support (Bey et al., 2019; Herriott et al., 2024; Kathawa et al., 2022; Maru et al., 2025; Schubert et al., 2024).

For example, a study involving 100 Black young mothers who received support from a community‐based doula found that doulas engaged closely with the mothers' family members (Herriott et al., 2024). Participants reported feeling safer during the birthing process and more empowered to advocate for themselves, particularly when medical procedures did not align with their preferences (Herriott et al., 2024). Similarly, in a qualitative study of 14 Black women who received community‐based doula support, Arteaga et al. (2023) found that participants reported feeling understood, supported, and emotionally prepared throughout pregnancy and childbirth. Notably, none of the participants described their births as negative, despite some reporting complications during childbirth (Arteaga et al., 2023). Collectively, these findings underscore the urgent need to address disparities in maternal mental health, particularly in the context of the ongoing United States (US) maternal health crisis, in which Black women continue to experience disproportionately high rates of maternal morbidity and mortality due to systemic racism and inadequate social support in clinical settings (Crear‐Perry et al., 2021; Suarez‐Balcazar et al., 2024).

Considering their unique and trusted relationship (Arteaga et al., 2023; Herriott et al., 2024), community‐based doulas might play a critical role in addressing or filling gaps in perinatal mental health care for their Black clients. Although there is growing recognition of doulas' involvement in identifying and supporting clients experiencing perinatal mental health and substance use challenges (Haiman et al., 2025; Quiray et al., 2024), the specific ways in which community‐based doulas engage with perinatal mental health and their openness to using formal, validated screening tools remain unclear. In particular, questions persist regarding their potential role in addressing perinatal anxiety and depression and the acceptability of engaging in formal mental health screening within their scope of practice. Therefore, gaining insight into community‐based doulas' perspectives on using formal, validated mental health screening tools is critical. Understanding their approaches to addressing perinatal mental health may inform preventive strategies and help expand access to equitable perinatal mental health care.

## Current study

Community‐based doulas are not responsible for dismantling structural inequities and systemic issues in perinatal mental health care. However, they are important stakeholders in the process (e.g., supporting community and traditional healing practices; shared decision‐making) of achieving equitable, antiracist mental health care (Matthews et al., 2021; Suarez‐Balcazar et al., 2024). In this study, we explored community‐based doulas' perceptions and beliefs regarding the acceptability of using formal, validated anxiety and depression screening tools to assess mental health among perinatal Black women. We investigated two key questions: (1) Are community‐based doulas receptive to utilizing validated mental health screening tools with their Black clients? (2) How do they perceive their role in addressing the mental health needs of this population?

## METHODS

To address disparities in access, effectiveness, uptake, and reach of perinatal mental health services for Black women, we used a transformative and pragmatic research approach (Mertens, 2007, 2009; Morgan, 2007). The transformative approach prioritizes the production of contextually relevant knowledge to advance social justice for historically marginalized communities (Mertens, 2007, 2009). It is inherently collaborative, valuing the experiences and insights of participants to inform and drive meaningful change (Mertens & Wilson, 2013). In alignment with this framework and to ensure the study reflected community priorities without imposing external screening practices, we actively engaged community‐based doulas. While they did not formally join the research team, they provided feedback on interview questions and disseminated recruitment materials through their networks. Their insight was instrumental in shaping this study, which aims not only to explore existing practices and perspectives but also to inform future collaborative efforts to promote perinatal mental health equity for Black women.

In addition, we incorporated a pragmatic approach to improve the screening and prevention of perinatal mental health disorders in this population. Pragmatism emphasizes practical, actionable solutions to real‐world problems by focusing on what works in clinical and community practice (Creswell & Clark, 2018; Morgan, 2007). A key feature of this approach is the use of targeted, applied questions to inform and improve interventions. Moreover, given the urgent need to enhance the detection and treatment of perinatal anxiety and depression among Black women, combining transformative and pragmatic approaches allowed us to establish a foundation for the development of future community‐informed interventions in this critical area.

### Researcher positionality

The principal investigator, Author T.N., is an African American clinical psychologist with a PhD in clinical psychology, an MPH in epidemiology, and postdoctoral research and clinical training in perinatal mental health. A mother of a tween, she brings extensive experience in community‐engaged, qualitative, and quantitative research. She has provided clinical care across diverse settings, including early childhood home‐based services to bridge gaps in access to mental health treatment for families who cannot afford it, community mental health, and the nation's first perinatal partial hospital program. Mindful of historical, systemic, and institutional injustices, she is committed to research and clinical care that are contextual, culturally responsive, and ethically grounded, prioritizing safety, respect, and community well‐being.

The second author, N.N.S., is an African American woman who recently earned her PhD in clinical psychology with a focus on behavioral medicine and health psychology. She is trained in qualitative research and brings over a decade of experience conducting quantitative and qualitative research with Black women in community and clinical contexts. As a clinician, she has a background in women's health, providing trauma‐informed care to women from historically marginalized backgrounds during the perinatal period. The third author, S.B.G., is an African, Ethiopian, cisgender woman and a graduate student whose identity and lived experiences as a Black immigrant shape her research focus and values, emphasizing cultural humility, equity, and representation. Over the past 4 years, she has worked as a research assistant in the first author's lab, studying mental health disparities, help‐seeking behaviors, and perinatal health among Black and immigrant populations. Currently, a research coordinator for community mental health initiatives, she facilitates focus groups, analyzes data, and engages in methodological training. Her experiences have reinforced her commitment to community‐driven research as a means of advocacy, visibility, and systemic change, and to amplifying the lived experiences and strengths of the communities she serves and belongs to. The fourth author, C.C.J., is a cisgender African American woman who recently earned her master's degree in psychology. Her research has focused on mixed‐methods studies exploring gendered stereotyping, racial identity, and the marginalization of communities of color. As a Black woman, her lived experiences have profoundly shaped her academic interests, particularly in the areas of inequality, prejudice, and stereotyping across interpersonal, community, and systemic levels. These experiences continue to fuel her commitment to advancing scholarship on the intersectional challenges faced by Black women. The fifth author, P.D.T., is a Caribbean American cisgender woman and PhD candidate in Prevention Science. A first‐generation doctoral student and former mental health therapist, she draws on personal and professional experience to address maternal health disparities in Black communities. Her understanding of trauma, systemic barriers, and mental health is shaped by both academic training and direct service. While sharing cultural and professional ties with many participants, she remains mindful of her institutional privilege. She approaches her work with cultural humility, centering participant voices and community feedback throughout the research process. The sixth author, K.S.A., identifies as an Afro‐Caribbean, cisgender woman who is a nurse scientist and researcher with a PhD and a Master's in Nursing. She has three adult children, one delivered by a physician, one delivered by a nurse‐midwife, and one unplanned home birth. She has extensive knowledge and expertise in both clinical and nonclinical aspects of perinatal care with parents across race, ethnicity, gender, geography, and economic status, with an emphasis on pregnant people who are most marginalized by systems of care. She has over 20 years of experience as a certified nurse‐midwife and an assistant professor whose research interests examine and explore the experiences of stress and psychological distress in Black perinatal people. In her role as Director of her own nurse‐midwifery practice within a larger OB/GYN practice, she focused on providing equitable care across the lifespan to those who are often the most overlooked and underserved. As a mixed‐methods researcher, she centers the lived experience of study participants to gain a greater depth and breadth of understanding of the contextual nuances of navigating the perinatal period within the current healthcare system and the complexities of bias, discrimination, structural racism, and interpersonal racism.

### Participants

The sample consisted of 30 community‐based doulas. Most participants identified as Black or Indigenous (92.5%). Forty percent were between the ages of 25–34; 43.3% were 35‐44, and 16.7% were aged 45 and older. Nearly all participants were born in the United States (*n* = 29) and ranged in years of practice from 1 to 14 years (*M* = 4.38, SD = 3.17). The average number of visits provided during the first, second, third trimester and postpartum period were 2.25 (SD = 1.36), 2.93 (SD = 1.51), 3.40 (SD = 2.08), and 3.37 (SD = 2.14), respectively.

### Measures

#### Demographic questions

Before the interview, we asked participants questions about their age group, race/ethnicity, place of birth, number of years in practice, and the number of visits they generally provided to their clients during each trimester. All community‐based doulas responded to demographic questions before the interview began.

#### Interview protocol

We asked community‐based doulas the following questions:

1. Do you screen for depression or anxiety with your clients?
2. Here are some mental health screening tools that have been validated for use during pregnancy and postpartum. [Show Edinburgh Postnatal Depression Scale (Cox et al., 1987), Patient Health Questionnaire‐9 (Kroenke et al., 2001), and Perinatal Anxiety Screening Scale (Somerville et al., 2015)] This means that researchers made sure that the items being asked were reliable and valid in detecting anxiety and depression. What do you make of these measures? Would you use them? Why/why not?
3. Are community‐based doulas able to address the mental health needs of Black pregnant and postpartum women they support? Why or why not? How?

### Procedure

The institutional review board (IRB) of the first author's institution approved this study. The first author conducted all semi‐structured interviews with 30 community‐based doulas aged 18 and over, serving perinatal Black women. We used convenience, referral‐based sampling to recruit community‐based doulas via email to local and national Black community‐based doula organizations. After obtaining informed consent, we collected demographic information via Qualtrics. We then introduced the Edinburgh Postnatal Depression Scale (EPDS; Cox et al., 1987), Patient Health Questionnaire‐9 (PHQ‐9; Kroenke et al., 2001), and Perinatal Anxiety Screening Scale (PASS; Somerville et al., 2015). We solicited participants' perceptions, thoughts, and beliefs regarding the acceptability of utilizing these tools to assess mental health in their perinatal Black clients and whether they engaged in shared decision‐making with their clients when utilizing these tools in their practice. All interviews were conducted in the preferred location and method of each community‐based doula. Interviews were held in person or via Zoom and audio recorded. Upon completing the study, participants received a $50 electronic gift card via email. Transcripts were professionally transcribed for data analysis. We used pseudonyms to protect the participants' confidentiality.

### Data analysis

We used thematic analysis to identify ideas (i.e., codes) and recurring patterns (i.e., themes) via a rigorous process of data familiarization, data coding, theme development, and revision (Braun & Clarke, 2006). The first and second authors actively read and discussed two transcripts to gain a deep understanding of the content. This initial exploration informed the development of a preliminary codebook by the second author. The first author then refined the codebook and independently developed initial themes based on participant responses, regardless of their stance on using screening tools. The first and second authors discussed the themes, understanding that additional themes might emerge during the analysis. To ensure consistency, both authors independently coded the interview data using NVivo 14, a qualitative data analysis software program (Lumivero, 2023). Following coding, we met regularly to discuss the fit of the data with themes (Levitt et al., 2017). We considered alternative interpretations of the interview data, acknowledging the potential for multiple perspectives, and resolved discrepancies through reflection and dialogue (Levitt et al., 2017; Patton, 2014).

## RESULTS

Most community‐based doulas (60%) reported that they did not screen their clients for perinatal anxiety or depression. Screenings, when performed, were typically conducted by nurses, social workers, or case managers during the intake process. Overall, most community‐based doulas supported screening to detect perinatal anxiety and depression. When asked if they would use any of the validated screening tools in their practice, 57% supported, 23% opposed, and 20% were unsure. Four themes underscored the acceptability of using validated screening tools in community‐based doula practice: (1) screening approaches and procedures; (2) cultural and contextual screening; (3) community‐based doula roles; and (4) client trust (see Table 1 for a summary of themes, codes, and representative quotes).

**Table 1:** Acceptability of screening for perinatal mental health disorders: themes, codes, and representative quotes.

Theme	Supporting codes	Representative quotes
Rethinking screening approaches and procedures	Ask different questions Baseline/Guideline Create safe space Early detection Listen Observe Post‐screening procedures Self‐administer Simplify language	“I would be comfortable asking everything. I wouldn't give them the paper. I probably would do it for them. I would just ask them the question and fill it out.” —Hadiyah
Cultural and contextual screening	Context Cultural lens Cultural norms Cultural nuances Culturally inappropriate Social Location/Position	“Yes, I think some sort of assessment needs to be done, but the existing assessments don't really do a good job at acknowledging the cultural nuances.” —Yvonne
Community‐based doula roles	Boundary recognition Clinical Research purposes Role identity/purpose	“To be honest, this brought up some stuff for me because I was just, I felt like this might be out of my league. If someone's dealing with something super deep, I'm not a psychologist. I don't really know what to do besides some mantras and breathing.” —Renee
Client mistrust of mental health questionnaires	Fear of Child Protective Services Fear of disclosure Medical providers mistrust Mental health stigma	“There's a very valid fear, especially if you're a Black woman, that something harmful can happen to your family if you disclose this information.” —Angela

### Acceptability of screening for perinatal mental health disorders

#### Rethinking screening approaches and procedures

Participants across all three groups (i.e., supportive, opposed, and unsure) expressed concerns regarding perinatal mental health screening approaches and post‐screening support. Discussions emerged around alternative screening methods, focusing on how conversations with clients could be used instead of simply administering a questionnaire. These discussions also addressed potential next steps following a screening, highlighting the importance of postscreening support. For example:

*I would be comfortable asking everything. I wouldn't give them the paper. I probably would do it for them. I would just ask them the question and fill it out. And then depending on what I gather, that the points match. [For example], I would say, “Because I know you've had to fill it out with your doctor too as well, are you telling me these things when you visit? And if you are, you might want to check in what resources they have, or you might want to look into those things just check in and make sure we're all good.” So yeah, that's probably how I would spin it*. –Hadiyah, 35‐44 years old, 3.5 years in practice

*I definitely don't know enough about the questionnaires to say which should be used, which shouldn't be used. My initial thoughts are I see people‐‐ I see providers use these all the time, and they sort of just go in the wastebasket, and I think people should be having real conversations and spending real time with patients. Right? Kind of like you did with the consent form, going through this with‐‐ you went through it with me, and we had an experience together about the consent form. You didn't hand me the consent form or email it to me beforehand, and then I ignored it and said I read it or whatever. Right? I think with a lot of these things, it's like having an actual dialogue about mental health, I think that's different. That is not how I've seen these administered. I think often, yeah, there's not a lot–it's kind of a scale that alerts a red alert. You know, “Red alert in the system, connect with another person.” This provider and this provider, not attached, who never talk to each other*. –Sarah, 25‐34 years old, 5 years in practice

Some participants reported providing referrals to therapy and expressed concerns about the next steps post‐screening, the referral process, and the client experience. For instance:

*I would like to do it with my clients, as long as they're open to it. But I think the thing about it is I would like to do it‐‐ I would be nervous about, okay, if I did it, and then, say, if I notice that they are‐‐ if they are screening very high, are they going to be open to the next steps of support and things like that? I think that that's the thing that I'm always interested to know, like, well, what would that look like, as far as after I'm done administering it?* –Fannie, 25‐34 years old, 5 years in practice

*I think these are great. And it's something that I kind of wish I would incorporate, especially when I do private practice because, I mean, just looking at this, I'm like, You know, this would be great. And if I had a therapist that I trusted that I could refer out to, that would be a very good tool for my client. And I think I can definitely get my client‐‐ because I spend a good amount of time with them where I could just pull this out and I can ask these questions on my own and check out the boxes*. –Gladys, 35‐44 years old, 6 years in practice

*I refer my clients to therapists, but these therapists are private insurance therapists. I also do refer them to general resources as well, but I can't say that I personally use those resources. So, I'm not even sure about the end‐user experiences. So, I can refer you, but if you can't afford it, how good is it?* –Miriam, 35‐44 years old, 2 years in practice

#### Cultural and contextual screening

Across decision points, community‐based doulas discussed culturally appropriate and contextual screening as important for their decision‐making. Responses generally emphasized the cultural relevance of the tools, cultural sensitivity, and context to detect perinatal anxiety and depression in Black clients. For example:

*It's annoying. It's technical, it's paper, and feels very clinical. It feels very European based. That's not how African Americans get down. They don't want to look at a paper and be like check, check, check. And don't get me wrong. I've filled it out before. But realistically speaking, I think it's more about a conversation*. –Bianca, 35‐44 years old, 10 months in practice

*Those measures, while they can be useful, they're not necessarily useful for us*. –Ella, 55‐64 years old, 5 years in practice

*I do think that they are beneficial, but I would want to know, how do they account for the cultural norms that someone may be experiencing? My thing is–depression looks different across cultures, but I also want to make sure that, maybe, it's the person that is administering the screening, and how do they look at it, from a cultural lens? So, to someone else, they may be like, oh that's anxiety, and things like that, and I'm like is it? Or is it because of an impact of something else? So, I think that they are beneficial, but I also think about the lens of the person that's administering it*. –Fannie, 25‐34 years old, 1 year in practice

*Yeah, this needs to be done–I feel like sometimes my work as a doula is filling in gaps that a person's provider hasn't filled with referrals or whatever. I also think it's a challenge that sometimes the rating scales and the things that people are told don't always match my client's signs and symptoms. Yes, I think some sort of assessment needs to be done, but the existing assessments don't really do a good job at acknowledging the cultural nuances*. –Yvonne, 25‐34 years old, 1.5 years in practice

#### Community‐based doula roles

Many community‐based doulas identified themselves as dual healthcare professionals, including nurses, social workers, therapists, and case managers. Nonetheless, several participants distinguished between their primary function as supportive figures and the professional role of a psychologist or mental health provider. For instance:

*[Screening]takes the, I guess, connection out of the experience. Like, I'm connecting with someone…I'm listening to them and hearing and understanding. I do ask them, like, do you sleep and are you hanging out with your friends? Are you getting out? Are you getting time away from the baby? But if I'm asking [using] that paper, I don't get to tell you like, “Yeah, you and babies need breaks from each other. That baby likes breaks from you.” And I'm not getting to be me and experience—like establish that relationship [so that] they want to reach back out to me and talk to me later on. And so, I feel like I wouldn't want to do that. I feel like that is definitely the role of a doctor*. –Iris, 25‐34 years old, 1 year in practice

*To be honest, this brought up some stuff for me because I was just, I felt like this might be out of my league. If someone's dealing with something super deep, I'm not a psychologist. I don't really know what to do besides some mantras and breathing*. –Renee, 35‐44 years old, 1 year in practice

Conversely, some community‐based doulas emphasized their pre‐existing relationships with clients and families as a rationale for conducting screenings in this population. For instance:

*I think the relationship that I have developed with my clients, if they experience any of those things, they pretty much tell me. But I also ask too. And I do visits and I observe. So the paper–I mean the questions are a good help if you're not able to consistently…I see why they exist*. –Bunmi, 25‐34 years old, 5 years in practice

*Because it does kind of‐‐ not to say become a blur or get swept under the rug, but even with a follow‐up with your doctor, sometimes us as women aren't as honest about what's really been going on within those 6 weeks. So, I feel like I would definitely use it. I mean, just because I feel like in general, clients will be more receptive to being more honest with their doula than their actual provider*. –Dorothy, 25‐34 years old, 5 years in practice

#### Client mistrust of mental health questionnaires

Participants expressed concerns about a potential rupture in client trust if they used screening tools. They worried that their clients might perceive screening as a judgment or questioning of their parenting abilities; and that clients might hesitate to reveal mental health problems due to a fear of losing custody of their children. For example:

*There's a very valid fear, especially if you're a Black woman, that something harmful can happen to your family if you disclose this information*. –Angela, 35‐44 years old, 6.5 years in practice

*You can get more out of a person without being technical. I think that there's a serious mistrust that our people have with the medical system. “Are you going to put me in here, and tell your supervisor I'm depressed?”* –Anna, 35‐44 years old, 6 years in practice

*A lot of people don't—they don't trust certain systems. They think that if they expose a problem within themselves, that somebody might be ready to take their child or somebody might be ready to have them committed…. But when they're coming to you and they're saying things to you that they're probably not going to share with anybody else, it doesn't matter—it's someone who lives near them, someone who sees and knows what they're going through. It's someone who looks like them and can identify with what they're going through. And in some cases, we are the best option to intercept when those problems arise*. –Bunmi, 25‐34 years old, 5 years in practice

*I think they'd definitely be helpful when meeting with clients and families. But, if I were to use these, I can't say that I'd be sitting there checking off each one; I feel like that could be intimidating. Oh, and this actually just made me think of one other challenge that I think comes up with mental health is fear that, if they are able to acknowledge that they're struggling with mental health, and they voice that to some professional, that it might seem like they're unfit. There might be a concern for child services involvement, especially as Black women. It really could be as–not saying it's a simple issue, but simple as you being depressed. If you express that to your pediatrician or OB, who's to say they're not going to overreact and report you?* –Lauryn, 25‐34 years old, 1 year in practice

*All this questionnaire stuff and then I'm an enemy to them*. –Zora, 65+ years old, 10 years in practice

### Addressing perinatal mental health needs

When asked if community‐based doulas could address the mental health needs of the Black pregnant and postpartum women they support, most (53%) responded “to a certain extent,” “it depends,” or “yes” and “no”; 30% said “yes”; and 17% said “no.” Four themes highlighted community‐based doulas' perceptions of how they might address perinatal mental health needs including identification and normalization of symptoms; bridge to mental health; systems issues; and mental health specialization. See Table 2 for a summary of themes, codes, and representative quotes.

**Table 2:** Addressing perinatal mental health needs: Themes, codes, and representative quotes.

Theme	Supporting codes	Representative quotes
Symptom identification & normalization	Empathize/validate Have conversations on mental health Normalize mental health symptoms Identify signs of mental distress Identify positive mental health Self‐disclosure—mental health Self‐disclosure—therapy	“I think we're able to start the conversation and help normalize… again, normalize, that's a word that [everybody uses?]. Normalizing the conversation because so many people are afraid to talk about it, or they think that it's just supposed to happen.” –Mae
Bridge to mental health	Attitudes supporting help‐seeking Attitudes supporting therapy Consult with mental health colleagues Encourage self‐care Provide mental health resources Provide therapy referral Involve partners if appropriate	“I think it depends on your education. Anyone could be a doula, so. Here I am with the whole background in emergency medicine. Is my specialty psychiatry? No, not at all. But I think that I build trusted relationships enough that if I was to say to someone, I really think that you should follow up with a therapist, that it would be a respected response.” –Sonia
Systemic issues	Hierarchy Inequities Insurance Lack of Black mental health providers Lack of funding/support Pay equity Structural Issues Systemic Issues Therapy inaccessible/unaffordable	“I want to say, “Yes and no.” Because I do think we play a role and can make an impact, in terms of education‐‐ referring to resources‐‐ being aware of the signs ourselves. But I don't think it falls on our shoulders solely. And it's a much—there's other people who need to be involved, and it's just a larger systemic issue other than just us.” –Lauryn
Mental health specialization	Educational/training opportunities Mental health doulas (specialty) Mental health toolkits for doulas Mental health training for doulas	“I think that there should be special training for it where the doula would only focus on that. So, like a mental health postpartum doula—something like that. I think with mental health on the rise, it has to be a specialty. Going into mental health is a specialty because you're dealing with the life of that person and you're dealing with the life of their children. That's heavier. So, I think it should be a specialty doula.” —Cicely

#### Identification and normalization of symptoms

The most salient theme of how community‐based doulas might address perinatal mental health with their Black clients centered on identification and normalization of symptoms. Participants described how the close relationship might help to destigmatize perinatal anxiety and depression in this population.

*If you mean identify, yes; I think the table is big enough for everybody. I think that doulas have a place in our healthcare system. And we have to figure out as an industry how we balance each other out. When you think about it, yes, a doula is not a medical professional. Right? But you have many doulas who have the background*. –Katherine, 35‐44 years old, 2 years in practice

*I think we are. And I definitely think that we are probably even more heightened and aware of what's going on with them because we are so close with them as far as in their care. So, unless you are just very far removed emotionally, you tap into that as a human being when you're talking to someone, and you can sense that something is just not right, right?* –Khadijah, 25‐34 years old, 5 years in practice

*I think we're able to start the conversation and help normalize…‐‐ again, normalize, that's a word that [everybody uses?]. Normalizing the conversation because so many people are afraid to talk about it, or they think that it's just supposed to happen*. –Mae, 25‐34 years old, 3 years in practice

#### Bridge to mental health

Several participants discussed providing referrals to trusted mental health care professionals. For example:

*We always scream, we're not clinical! Address them? I mean I might be able to point out…like, “I don't know honey, you might need to talk to someone. My suggestion is for a mental health professional to be included in the care team*. –Hadiyah, 35‐44 years old, 3.5 years in practice

*That is a big one. I think we can to an extent. I think professionally, we can't because we don't have the license…we can help by being very supportive. I'm also going to be an advocate for them to express themselves and to get help if they need it, and don't feel ashamed of it. I think that when it comes time to them wanting something done professionally, that's when we have to, of course, get them another resource*. –Mercy, 35‐44 years old, 1 year, 5 months in practice

*I think it depends on your education. Anyone could be a doula, so. Here I am with the whole background in emergency medicine. Is my specialty psychiatry? No, not at all. But I think that I build trusted relationships enough that if I was to say to someone, I really think that you should follow up with a therapist, that it would be a respected response*. –Sonia, 35‐44 years old, 6 years in practice

#### Systemic issues

Beyond highlighting systemic issues in the mental health care system, participants acknowledged the limitations of their role. They expressed concern that community‐based doulas cannot be the sole solution for addressing the perinatal mental health treatment needs of their Black clients. For example:

*We can't do it. I'm only one doula. There's a whole medical industrial complex that this person is a part of. I'm just one touchpoint…I can't possibly address everything. This kind of support needs systematic integration and it needs systematic support, but support in a way that's not punitive. If more people didn't feel that they were going to be penalized for getting support, they would reach out*. –Angela, 35‐44 years old, 6.5 years in practice

*I want to say, “Yes and no.” Because I do think we play a role and can make an impact, in terms of education‐‐ referring to resources‐‐ being aware of the signs ourselves. But I don't think it falls on our shoulders solely. And it's a much—there's other people who need to be involved, and it's just a larger systemic issue other than just us*. –Lauryn, 25‐34 years old, 1 year in practice

*Are we able to? I mean, I'm sure we can, but is it our responsibility? I think that's like the biggest thing. And I was actually talking to a few of my doula friends and my midwife friends about this because a lot of what the media has been talking about is, oh, yes, doulas, specifically Black doulas and black midwives are the answer to this problem. No, that's the band‐aid, we're a part of the solution, but I think when we're thinking about maternal mental health and we're thinking about maternal morbidity and maternal mortality, it's a systemic issue. It's not and I think a lot of times people say, oh, yes, there's not enough representation, which is 100% true, and I agree with that. I think I said that earlier. But by putting more black women or black men or just black people in general in these positions, it's not necessarily going to change the problem if the system is broken*. –Nnenna, 25‐34 years old, 6 years in practice

#### Mental health specialization

Participants also recommended specialized mental health training for doulas, emphasizing the importance of culturally sensitive communication when discussing mental health with perinatal Black clients. For instance:

*I think that there should be special training for it where the doula would only focus on that. So, like a mental health postpartum doula—something like that. I think with mental health on the rise, it has to be a specialty. Going into mental health is a specialty because you're dealing with the life of that person and you're dealing with the life of their children. That's heavier. So, I think it should be a specialty doula*. —Cicely, 45‐54 years old, 14 years in practice

*I think we are just scratching the surface of trying to expose people to mental health. And let them know that it's real…I think just like medical professionals have continuing medical education, I think that doulas should have it too. It shouldn't be, okay, you're a doula, good luck, type of thing. I think these things are happening in our community. We're going to talk about them. So that we can speak to them and speak to them the right way, not like, girl, you crazy. You need to go talk to somebody. Like, no; not like that*. —Maya, 45‐54 years old, 9 years in practice

## DISCUSSION

Despite recommendations for universal screening, perinatal anxiety and depression screening among Black women is low (Declercq et al., 2022; Geier et al., 2015; Lara‐Cinisomo et al., 2018; Sidebottom et al., 2021). We explored community‐based doulas' perspectives and beliefs utilizing validated screening tools for perinatal anxiety and depression, and how they might approach addressing the mental health concerns of Black women. Consistent with previous research on traditional doulas (e.g., Quiray et al., 2024), findings indicate some community‐based doula buy‐in for conducting formal perinatal anxiety and depression screening with their clients. Notably, over half of the participants (57%) supported screening and outlined key factors for successful implementation in partnership with community‐based doulas. Screening perspectives revolved around four key areas: (1) appropriate screening approaches; (2) the cultural appropriateness of the screening tools; (3) the role of community‐based doulas in the process; and (4) the potential impact, both positive and negative, that screening might have on the client‐doula relationship. Themes capturing community‐based doulas' role in addressing clients' mental health needs included: (1) identification and normalization of symptoms; (2) referral to treatment; (3) systems issues; and (4) mental health specialization.

In the theme, *rethinking screening approaches and procedures*, community‐based doulas described administering screening tools themselves and having a dialogue around mental health rather than having clients complete them independently. This is important as screeners are not diagnostic, and the standard recommendation is to engage in a conversation about the responses on the questionnaires. Though mostly supportive of screening, some community‐based doulas felt that their clients needed assistance understanding the process to understand its purpose and results. Our findings align with qualitative research on patient perception of perinatal depression screening, where participants reported feeling disconnected from the screening process due to inadequate discussion with healthcare providers, a lack of explanation about the purpose of the screening, and no follow‐up on results (Hsieh et al., 2021). In another study, Black Caribbean women in the United Kingdom described having minimal time to discuss their mental health concerns with their providers, noting, “leaflet (baby massage); leaflet (postnatal depression); leaflet (baby immunization). Any questions let us know. Any problems see your GP. See you later” (Edge, 2011). This quote demonstrates how some consultations don't allow enough time to discuss their mental health concerns, which can lead to disengagement, which, in turn, can diminish the overall effectiveness of perinatal anxiety and depression screening and referral treatment (Hsieh et al., 2021).

Disengagement from the screening process warrants increased attention to strategies that enhance screening effectiveness and subsequent referral and treatment pathways for perinatal anxiety and depression in this population. Many Black women who screen positive for perinatal depression lack adequate referrals and follow‐up care, hindering their access to proper treatment (Mestad et al., 2016). Our findings suggest the need for additional community‐based approaches that include strong collaboration between community‐based doulas and trusted, culturally responsive mental health professionals. For example, programs like Warm Connections, which utilize the Special Supplemental Nutrition Program for Women, Infants, and Children (WIC) staff to screen clients using the EPDS and directly connect them to treatment resources, demonstrate the effectiveness of community‐based screening with immediate referral pathways (Klawetter et al., 2020). Future prevention efforts might adapt and research this model to determine if it results in increased detection and treatment of perinatal anxiety and depression among Black women.

Despite validation for detecting perinatal anxiety and depression in the general population, some community‐based doulas raised concerns about the cultural appropriateness of screening tools with their clients. The theme, *cultural and contextual screening*, revealed participants' perceptions that screening might be limited in their ability to capture symptoms of distress experienced by their Black clients. Research suggests that the EPDS is a reliable measure for use with perinatal Black women, although lower cut‐off scores may be necessary to accurately detect depression (Tandon et al., 2012). However, it is unclear if the PHQ‐9 has been validated for use with Black women during pregnancy, despite its application in research studies and clinical settings with this population (Sidebottom et al., 2012, 2023; Wenzel et al., 2022). As a relatively new screening tool, the PASS, while used clinically, has not yet been validated in this population. This highlights the need for additional research validating perinatal anxiety screening tools for use with this population.

The theme, *community‐based doula roles*, reflected how some community‐based doulas expressed hesitation about screening, noting its “clinical” nature and believing it to be a better fit for psychologists or doctors. While the roles of community‐based doulas as nonclinical support persons are well documented, the role of lay health professionals as a “bridge” between the communities and formal systems of care is also well established (Ayala et al., 2010; Barnett et al., 2018). Lay health professionals' models of care have championed a four‐pronged approach to reduce disparities in mental health services: increasing awareness, decreasing stigma, conducting screenings, and providing treatment referrals (Barnett et al., 2018). Importantly, we found that many community‐based doulas in this sample were already identifying perinatal Black clients' experiences of mental health symptoms and facilitating referrals for treatment. This provides further evidence that community‐based doulas not only provide collaborative support alongside family, friends, and healthcare providers but also fill gaps in healthcare (Arteaga et al., 2023; Herriott et al., 2024; Maru et al., 2025; Schubert et al., 2024). Therefore, strong partnerships with mental health professionals to provide training, consultations, and clear referral pathways might enhance community‐based doulas' efforts in this capacity. However, concerns about the relationship remain, as some community‐based doulas contemplated the potential impact of screening on their clients.

Findings emphasized the potential negative impact of perinatal anxiety and depression screening on the client‐doula relationship. The theme, *client mistrust about mental health questionnaires*, highlighted the tension between the trusting relationships that community‐based doulas build and how those bonds might be undermined by perinatal anxiety and depression screening. For example, one participant noted that screening would “make her an enemy” to her Black clients. Other community‐based doulas described how their Black clients might fear over‐pathologization or losing their children if they report symptoms of anxiety or depression. This finding is consistent with McComish et al. (2013), who found that participants preferred materials framed around “adjusting to motherhood” rather than postpartum depression, due to fears that disclosing depressive symptoms could lead to being seen as “crazy” and risk losing custody of their child (McComish et al., 2013, p. 6). Moreover, this concern stems from historical and ongoing racial biases in the healthcare system and is directly connected to the current Black maternal health crisis (Clarke et al., 2022; Crear‐Perry et al., 2021; Declercq et al., 2022; Kathawa et al., 2022; Matthews et al., 2021; Mehra et al., 2020; Rosenthal & Lobel, 2011, 2020; Suarez‐Balcazar et al., 2024). It also underscores Black women's experiences of gendered racialized pregnancy stigma and pregnancy‐specific stress, which is associated with stress (Clarke et al., 2022; Keefe et al., 2017, 2018; Mehra et al., 2020; Rehbein et al., 2024; Rosenthal & Lobel, 2011, 2020) and with adherence to Superwoman Schema, a gendered racialized coping response, which may exacerbate perinatal anxiety and depression (Nelson, Tomi, et al., 2023; Woods‐Giscombé, 2010).

Participants' perspectives on addressing mental health varied. While only 30% affirmed that community‐based doulas could support the mental health needs of their Black clients, a greater proportion (53%) expressed mixed views indicated by “yes and no” responses. Key areas for intervention included: identifying and normalizing symptoms; bridging to mental health treatment; systemic issues; and advocating for community‐doula‐based certification in mental health. Despite mixed responses, our findings indicate that some community‐based doulas already view informal perinatal mental health screening as acceptable, appropriate, and aligned with their existing support for Black clients. The themes, *identification and normalization of symptoms*, and *bridge to mental health*, suggest that community‐based doulas possess valuable skills that can be further developed through training and support to become more effective in this area. The theme of *mental health specialization* further emphasizes this sentiment, suggesting possibilities for formalized training and certification. This could open doors for partnerships with mental health professionals to provide additional support. Nonetheless, additional research is needed to explore the feasibility and effectiveness of integrating formal screening tools and mental health training into community‐based doula practices.

While our findings highlight potential areas for intervention, we must acknowledge the systemic challenges in our mental health care system. These include limited access to mental health services, unequal treatment, structural inequities, and discrimination that place perinatal Black women at increased risk for anxiety and depression (Crear‐Perry et al., 2021; Lara‐Cinisomo et al., 2018; Suarez‐Balcazar). Additional challenges include community‐based doulas' experiences of gendered racism, stigma, and power differentials in spaces (e.g., hospitals, clinics, training) where they support their Black clients (Adams & Curtin‐Bowen, 2021; Thomas et al., 2023). Accordingly, community‐based doulas cannot single‐handedly dismantle structural inequities, nor should they be expected to “save the day” as several participants in the current study noted. However, their work is a crucial piece of the puzzle. For example, a recent study found that community‐based doulas in one program spent an average of 32 h supporting their clients, much of which is beyond direct care (Arcara et al., 2023). Thus, we implore scholars and clinicians to develop culturally informed interventions and training programs *in collaboration* with community‐based doulas. Further, policymakers and administrators should ensure that community‐based doulas are proportionately compensated for the tremendous amount of time, effort, and services they provide.

### Limitations

This study has several limitations. First, this sample included community‐based doulas located in the northeast region of the United States. Community‐based doulas in different geographic regions may hold different perspectives on this issue. Further, as this is a qualitative study, results may not generalize to the larger population of community‐based doulas working with perinatal Black women. Second, we included a purposive convenience sample of community‐based doulas, which raises concerns about selection bias, as readily available doulas might hold specific viewpoints on screening. Third, many participants reported practicing in various settings such as hospital‐based doula programs, community‐based doula programs, and independent practice, organically, without being prompted. Several participants also described full‐time employment as case managers, social workers, nurses, and allied health professionals. However, we did not inquire about the participants' professional backgrounds and locations of community‐based doula practice as part of our interview process. As a result, the lack of uniform data limits our understanding of how these factors might influence their perspectives on screening. Future research should explore the intersection of community‐based doulas' roles as both allied health professionals and community members and investigate how this dual identity influences their perspectives on mental health screening and referral to treatment.

## CONCLUSION

In this study, community‐based doulas provided invaluable insight for addressing Black perinatal mental health. Findings might be used to help facilitate screening protocols and approaches that may aid in effectively identifying perinatal Black women at risk for anxiety and depression and referring them to treatment. Community‐based doula voices and perspectives are critical to intervention and prevention efforts with perinatal Black clients. We recommend that researchers and organizations establish meaningful partnerships with community‐based doulas before developing protocols and designing interventions to address mental health. Finally, we reinforce the call by scholars for community‐based doula care to be recognized as a skilled profession with fair compensation; integrating doula services into healthcare and insurance systems, especially to promote health equity, requires acknowledging this expertise (Arcara et al., 2023).

## AUTHOR CONTRIBUTIONS

**Tamara Nelson**: Conceptualization, investigation, software, formal analysis, methodology, writing. **Naysha N. Shahid**: Software, formal analysis, validation, writing—review and editing. **Samrawit B. Gebretensay**: Validation, writing—review. **Chareina C. Johnson**: Writing—review. **Penny D. Telesford**: Writing—revision and review. **Karen Sheffield‐Abdullah**: Writing—review and editing.

## CONFLICT OF INTEREST STATEMENT

The authors declare no conflicts of interest.

## ETHICS STATEMENT

All research described herein has been conducted according to the American Psychological Association's ethical guidelines and has been approved by the Institutional Review Board of Rutgers University. Informed consent was obtained from all participants in this study.
